# Relationship of Vitamin B12 Levels With Different Degrees of Obesity and Diabetes Mellitus

**DOI:** 10.7759/cureus.47352

**Published:** 2023-10-19

**Authors:** Filiz Mercantepe

**Affiliations:** 1 Endocrinology, Diabetes and Metabolism, Faculty of Medicine, Recep Tayyip Erdogan University, Rize, TUR

**Keywords:** nutrition and metabolism, obesity, diabet melitus, serum vitamin b12, aging

## Abstract

Introduction:The potential influence of micronutrient status on obesity should be considered. Nevertheless, previous research examining the relationship between serum vitamin B12 levels and obesity has yielded inconclusive results. The objective of this study was to investigate the associations between serum vitamin B12 levels and obesity and diabetes mellitus (DM) in a population consisting of persons aged 18 years and older.

Methods: A retrospective case-control research was undertaken on a sample of 1024 individuals aged 18 years and older who were admitted to a tertiary healthcare facility (Recep Tayyip Erdoğan University Education and Research Hospital, Rize) for either overweight-related issues or routine check-ups. The primary objective of this study was to assess the B12 levels of these individuals. The researcher recorded the body mass index (BMI) and history of DM for all subjects.

Results: The study comprised a total of 1024 participants, consisting of 834 females and 190 males. The levels of vitamin B12 in women were found to be 308±113 pg/mL, while in men, the levels were 304±125 pg/mL. The results of the statistical analysis indicate that there is no statistically significant disparity in vitamin B12 levels between males and females (p=0.748). There was a statistically significant positive correlation seen between age and B12 levels; however, the magnitude of this connection was found to be minor (p=0.000, R2=0.017). The study findings revealed that out of the 1,024 individuals evaluated, 179 individuals exhibited B12 levels below 200, while 845 individuals displayed vitamin B12 levels above 200. The study findings indicated that there was no statistically significant distinction observed in the occurrence of obesity and DM in relation to vitamin B12 deficiency (p = 0.938, p = 0.08, respectively).

Conclusion: The results of this study offer empirical support for the notion that there is no significant difference in vitamin B12 levels between individuals afflicted with obesity and diabetes and those unaffected by these conditions. Interestingly, it was shown that serum B12 levels exhibited a modest increase with advancing age.

## Introduction

Obesity, which is defined as the excessive accumulation of body fat, presents considerable health hazards and has emerged as a significant issue in public health on a global scale [1]. The genesis of this condition is complex and involves a combination of genetic, behavioral, metabolic, and environmental variables. The condition of obesity is widely recognized to be associated with a wide range of negative health consequences, including but not limited to cardiovascular illnesses and metabolic syndromes [2,3]. Within the intricate web of interrelated elements, there has been a growing scholarly interest in exploring the potential impact of micronutrient status, specifically focusing on vitamin B12 [4,5].

Vitamin B12, also known as cobalamin, is a crucial water-soluble vitamin that plays a fundamental role in various physiological processes, such as DNA synthesis, erythropoiesis, and the maintenance of brain integrity [6]. A deficiency in vitamin B12, primarily acquired from the consumption of animal-derived foods, results in a wide range of clinical manifestations, including exhaustion, anemia, and significant neurological deficits [4]. The significant involvement of vitamin B12 in conjunction with other B vitamins in the metabolic process of homocysteine, an amino acid that has established connections with cardiovascular risks, is particularly noteworthy [7,8].

Recent studies have indicated a possible association between the status of vitamin B12 and obesity [4,9]. Preliminary cross-sectional studies have indicated that individuals who are obese tend to exhibit lower levels of serum B12 compared to those who have a normal weight [3,10,11]. Multiple explanations have been proposed in order to elucidate this fact [3,6]. Bariatric surgical treatments, commonly utilized as an intervention for obesity, have the potential to result in impaired absorption of vitamin B12 [12]. On the other hand, it is plausible that the dietary patterns of persons who are obese may exhibit qualitative distinctions, hence resulting in diverse levels of B12 consumption [13]. Moreover, the modified metabolic environment in individuals with obesity may have an effect on the distribution, storage, and availability of vitamin B12 [9]. Furthermore, it is possible that obesity per se could impact the allocation and retention of vitamin B12 inside the body, hence reducing its bioavailability [3]. One noteworthy component of this association is the potential existence of a feedback loop: while obesity has the potential to result in decreased amounts of vitamin B12, inadequate levels of B12 may also contribute to difficulties associated with obesity [3]. One example is the potential association between deficient levels of vitamin B12 and increased levels of homocysteine, which may contribute to the development of insulin resistance and other metabolic dysfunctions frequently observed in individuals with obesity [14]. Nevertheless, the existing body of literature does not provide a unanimous consensus. While several research studies emphasize the association between a deficit in vitamin B12 and obesity, other investigations fail to identify a significant correlation between these two factors [4,15,16]. These disparities may emerge due to diverse research methodologies, variations in study populations, and variances in the assessment techniques employed for evaluating both vitamin B12 levels and obesity.

In brief, the correlation between vitamin B12 and obesity, although substantiated by various research, remains complex and not entirely comprehended. Additional investigation is necessary in order to comprehend the underlying mechanisms of this correlation, its clinical ramifications, and the feasibility of utilizing B12 supplementation as a treatment approach for obesity and its related conditions.

The aim of this study is to investigate the effects of obesity, diabetes mellitus (DM), and age on vitamin B12 levels and additionally, to clarify the relationship between the vitamin B12 level and both obesity and DM, which is still discussed in the literature.

## Materials and methods

The present retrospective study was conducted at the outpatient clinic of the Division of Endocrinology at Recep Tayyip Erdoğan University Education and Research Hospital between October 2022 and May 2023. The study aimed to investigate the levels of vitamin B12 in adult individuals aged ≥ 18 years who sought medical attention for overweight or routine check-up purposes. The retrospective analysis involved examining age, gender, height, and body weight data. The relevant clinical data for this study was obtained through a review of medical electronic records registered in the national health database. The research protocol has been approved by the Local Ethics Review Board of Recep Tayyip Erdogan University. The work was conducted in accordance with the guidelines outlined in the Helsinki Declaration.

Patients and participants

The center where the study was conducted is a tertiary healthcare institution. A total of 1024 participants, consisting of 834 females and 190 males, were included in the study. Pregnant women, lactating women, those with a body mass index (BMI) below 18kg/m^2^ to exclude potential malabsorption, those diagnosed with known malabsorption disorders, individuals who have undergone bariatric surgery, cancer patients, individuals using metformin, and those undergoing vitamin B12 treatment were excluded from the scope of the study. Total vitamin B12 measurement Architect i2000 SR Chemiluminescent Microparticle Immunoassay (CMIA) on autoanalyzer (Abbott, Germany) worked with the method. Serum vitamin B12 measurements are given in picogram per milliliter (pg/mL). The results of vitamin B12 were evaluated based on gender, age, obesity, and BMI ranges. The calculation of BMI was performed by dividing the weight in kilograms by the square of the height in meters. Vitamin D levels were examined in female (n = 834) and male (n = 190) individuals to be compared in terms of gender. In addition, all participants were divided into two groups based on their BMI, namely BMI<30kg/m^2^ (non-obese, n=274) and BMI ≥30kg/m^2^ (obesity, n=750), in order to compare the levels of B12 vitamin in both groups. In order to evaluate patients according to BMI subgroups, they were divided into five groups based on BMI ranges: Group 1 (n=73) with BMI 18-25kg/m^2^, Group 2 (n=201) with BMI 25-29.9kg/m^2^, Group 3 (n=485) with BMI 30-39.9kg/m^2^, Group 4 (n=232) with BMI 40-49.9kg/m^2^, and Group 5 (n=33) with BMI >50kg/m^2^. Furthermore, the participants were categorized based on whether they had DM or not. Vitamin B12 levels of participants with DM (n=230) and without DM (n=794) were compared.

Statistical analysis

The statistical program utilized for all analyses was IBM SPSS Statistics for Windows, Version 22 (Released 2013; IBM Corp., Armonk, New York, United States). The variables were examined using visual and analytical techniques, including the skewness and Kurtosis Tests, to ensure their adherence to a normal distribution. The data exhibited a normal distribution. The mean and standard deviation (SD) were used to represent continuous variables that followed a normal distribution. The frequency and percentage were used to report the categorical variables. The application of the student’s t-test was limited to continuous variables that followed a normal distribution. The Tukey test with Bonferroni correction was utilized to do pairwise comparisons. The Pearson correlation coefficient was employed to conduct the correlation analysis. A p-value less than 0.05 was considered to be statistically significant.

## Results

**Figure 1:**
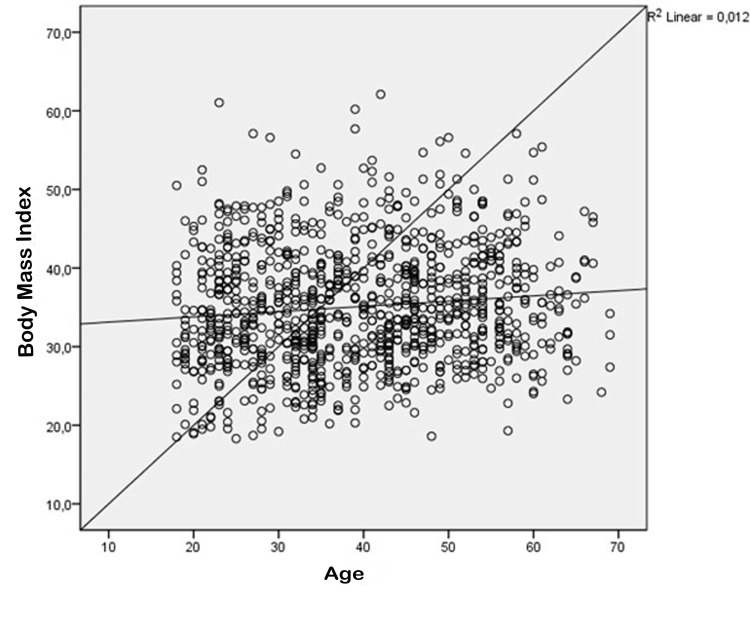
Correlations between BMI (body mass index) and age

General characteristics of the study participants are shown in Table 1. The research encompassed a total of 1024 individuals, with a median age of 39.5±12.5 years. This group consisted of 834 women, accounting for 81.4% of the participants, with a median age of 40±12 years. Additionally, there were 190 men, making up 18.6% of the sample, with a median age of 39±13 years. There was no significant age disparity observed between genders based on statistical analysis (p=0.228, Table 2). Out of the individuals who were part of the study, it was found that 794 individuals, accounting for 77.5% of the sample, did not possess a diagnosis of DM. Conversely, 230 individuals, representing 22.5% of the sample, had received a diagnosis of DM. There was a statistically significant difference in age between individuals diagnosed with DM and those without (p<0.001, Table 3). The study population was categorized into two groups: individuals who were classified as obese (BMI<30kg/m^2^, n=274, 26.8%) and those who were classified as nonobese (BMI≥30kg/m^2^, n=750, 73.2%). There was a statistically significant difference in age between individuals who were obese and those who were not (p<0.001, Table 2). Furthermore, the body mass index (BMI) of women (35.9±7.7kg/m^2^) was found to be significantly higher than that of men (31.5±6.2kg/m^2^) (p=0.000, Table 2). Additionally, a statistically significant positive correlation was observed between BMI and age (p=0.001, R2=0.012, Figure 1).

**Table 1:** General characteristics of the study participants (n = 1024)

Parameters		Total (n)	%
Participants		1024	100
Female		834	81.4
Male		190	18.6
Age (years), mean ± standard deviation		39.5±12.5	
Female		40±12	
Male		39±13	
Diabetes mellitus (DM)			
Yes		230	22.5
No		794	77.5
Obesity Status			
BMI<30kg/m^2^		274	26.8
BMI≥30kg/m^2^		750	73.2
BMI Categories			
18-24.9kg/m^2^		73	7.1
25-29.9kg/m^2^		201	19.6
30-39.9kg/m^2^		485	47.4
40-49.9kg/m^2^		232	22.7
>50kg/m^2^		33	3.2
Abbreviations: BMI, Body Mass Index			

**Table 2:** Relationship between age, BMI (body mass index), and B12 vitamin levels with sex *p<0.05 is significant

	Female (n=834)	Male (n=190)	p	Total (N=1024) (Mean ± SD)
Age (years)	39.6±12.3	38.4±13.3	0.228	39.4±12,5
BMI (kg/m^2^)	35.8 ± 7.6	31.5 ± 6.2	0.000*	35±7,6
B12 vitamin (pg/mL)	308±112	305±125	0.748	307± 115

**Table 3:** Age and vitamin B12 levels in DM and obesity *p<0.05 is significant DM, diabetes mellitus; BMI, body mass index.

		Age (years)(mean±SD)	p	Vitamin B12 (pg/mL)	p
DM	Yes (n=230)	42.4±13	0.000*	295±119	0.072
	No (n=794)	38.6±12.2		311±113	
Obesity (BMI≥30kg/m^2^)	Yes (n=750)	40.3± 12.4	0.000*	304±114	0.236
	No (n=274)	37.1±12.5		314±117	

**Figure 2:**
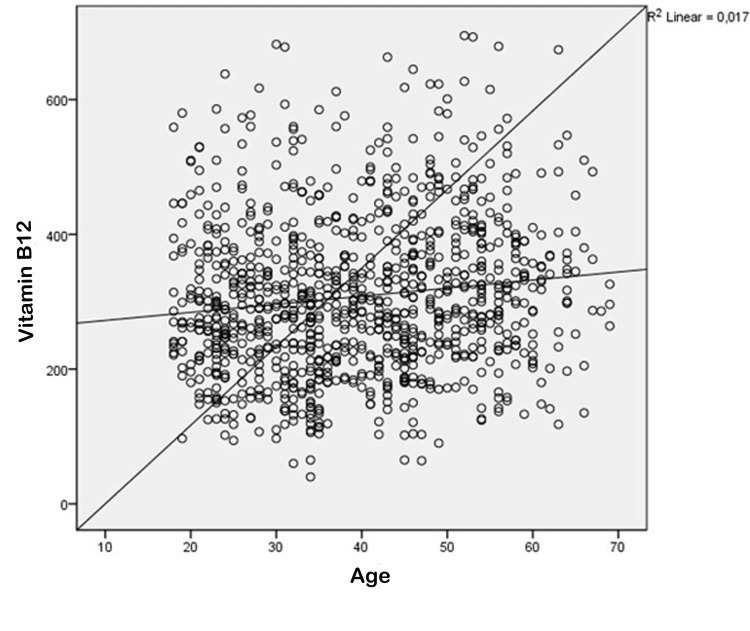
Correlations between vitamin B12 levels and age

In general, the study observed that the median levels of vitamin B12 were 307.5±115 pg/mL. Specifically, women had median levels of 308±113 pg/mL, while men had median levels of 304±125 pg/mL. The statistical analysis revealed that there was not a significant difference in the levels of Vitamin B12 between females and males (p=0.748, Table 2). A statistically significant positive correlation was seen between age and B12 levels but with a small effect size (p=0.000, R2=0.017, Figure 2). In order to ascertain the presence of vitamin B12 insufficiency, a cut-off value of 200 pg/mL was utilized. The study observed that out of the 179 individuals examined, their B12 levels were below the threshold of 200 pg/mL. Conversely, among the 845 individuals included in the study, their B12 levels were found to be over the threshold of 200 pg/mL. Upon comparing these groups in relation to their BMI, it was found that there was no statistically significant disparity observed between them (p = 0.456, Table 4).

**Table 4:** Relationship between vitamin B12 and BMI subgroups *p<0.05 is significant Abbreviations: BMI, Body Mass Index.

BMI (kg/m^2^)		B12 (mean±SD) (pg/mL)		P		
<25 (n=73)		324±127		0.632		
25-29.9 (n=201)		311±113				
30-39.9 (n=485)		304±117				
40-49.9 (n=232)		308±108				
≥50 (n=33)		294±116				
Vitamin B12 (pg/mL)	BMI ( mean± SD) (kg/m^2^)				P	
<200 (n=179)	34.7 ±6.9				0.456	
≥200 (n=845)	35.1±7.75					

The levels of vitamin B12 were measured in the DM group (mean±SD: 296±120 pg/mL), the non-DM group (mean±SD: 311±114 pg/mL), the obese group (mean±SD: 314±117 pg/mL), and the non-obese group (mean±SD: 305±115 pg/mL). Statistical analysis revealed no significant difference between the groups (p=0.08, p=0.938, respectively, Table 3). The correlation study did not yield any significant association between BMI and B12 levels. The participants were assessed based on their BMI subgroups, which were categorized as follows: BMI 18-25kg/m^2^ (n=73, 7.1%), BMI 25-29.9kg/m^2^ (n=201, 19.6%), BMI 30-39.9kg/m^2^ (n=485, 47.4%), BMI 40-49.9kg/m^2^ (n=232, 22.7%), and BMI >50 kg/m^2^ (n=33, 3.2%). The group with a BMI ranging from 25-29,9 kg/m^2^, referred to as group 2, exhibited the highest levels of vitamin B12 (Table 4). Conversely, the group with a BMI ranging from 40-49,9 kg/m^2^, referred to as group 4, had the lowest levels of vitamin B12 (Table 4). Nevertheless, there was no statistically significant difference observed in relation to the levels of vitamin B12 among any of the groups (Table 4).

## Discussion

The findings of this study indicate that there is no significant disparity in vitamin B12 levels between those who are obese and diabetic, and those who are not fat and non-diabetic. This conclusion presents an intriguing contrast to the existing body of literature, which has yielded inconclusive outcomes in terms of its alignment with prior research [17-19]. The results of our study align with prior research that has also seen no statistically significant alteration in B12 levels based on obese status [16]. Aroda asserted that there is no significant correlation between vitamin B12 levels and obesity [20]. Nevertheless, our research and its results contradict the prevailing belief that obese persons, who often experience significant metabolic changes, may possess varying levels of vitamin B12 [3,4,19,21,22].

Vitamin B12 (B12; also known as cobalamin) is a B vitamin that has an important role in cellular metabolism, especially in DNA synthesis, methylation, and mitochondrial metabolism [3]. Clinical B12 deficiency with classic hematological and neurological manifestations is relatively uncommon. However, subclinical deficiency affects between 2.5% and 26% of the general population depending on the definition used, although the clinical relevance is unclear [7,22]. B12 deficiency can affect individuals of all ages, and dietary intake of B12-containing animal-derived foods is restricted [4]. Deficiency is caused by either inadequate intake, inadequate bioavailability, or malabsorption [23]. Disruption of B12 transport in the blood or impaired cellular uptake or metabolism causes an intracellular deficiency [3,12,24]. Recently, numerous theories have been posited with respect to the potential ramifications of obesity and diabetes on vitamin B12 levels [3]. The impact of diabetes drugs, including metformin, on B12 absorption has been well acknowledged [25,26]. However, prior studies have shown inconsistent findings about the specific influence of diabetes medications, such as metformin, on B12 levels [11,26,27]. Nevertheless, individuals who were prescribed metformin were not included in the scope of this research. In the present investigation, no significant association was seen between B12 levels and the presence of DM. Unlike previous studies, the reason why there is no difference between B12 levels and obese or diabetic patients is that the patients we included in our study are patients who do not use metformin. Therefore, we think that the result of our study is more accurate.

With respect to age, a modest positive connection was seen between levels of vitamin B12 and advancing age. This finding presents a divergence from prior research, which suggests that the absorption of vitamin B12 diminishes as individuals age as a result of decreased production of stomach acid and other gastrointestinal alterations associated with aging [24,28,29]. Nevertheless, the presence of a modest positive association implies that the influence of individual factors, such as the decline in stomach acid production associated with aging and the rise in atrophic gastritis prevalence, is likely constrained. It is plausible that other variables, such as dietary patterns or disparities in lifestyle choices, might exert a notable influence. Nevertheless, it is important to consider that the perception of B12 deficiency being more prevalent among older individuals may not be entirely accurate. This is due to the potential influence of higher consumption of vitamin B12 supplements among the elderly or a diet that includes a greater proportion of fortified foods.

The findings of this study indicate that there is no statistically significant association between obesity and diabetes and vitamin B12 levels. Vitamin B12 levels were found to be comparable among those with obesity and diabetes, and those without these conditions. This contradicts prevailing notions that fat may impact the absorption of vitamin B12 or that diabetes may exert an adverse influence on B12 metabolism. A modest positive connection has been identified between age and levels of vitamin B12. This assertion is incongruous with the prevailing consensus that the absorption of vitamin B12 tends to decline as individuals age. Nevertheless, it is important to acknowledge that several circumstances, such as the augmented consumption of B12 supplements or alterations in dietary patterns over time, can exert an influence on this particular association. This study observes that there is no direct association between vitamin B12 levels and obesity or diabetes status. However, it does indicate that vitamin B12 levels tend to be higher with older age. Subsequent investigations ought to validate these findings within more extensive cohorts and explore additional plausible variables that could potentially impact vitamin B12 concentrations.

It is important to take into account both the strengths and flaws of our study. The study has notable strengths, including a substantial participant sample size and a meticulous application of inclusion and exclusion criteria. One of the study's shortcomings is its retrospective and cross-sectional design. Secondly, the relatively young age of the patients included in the study may have affected our results. The study identified a modest positive correlation between vitamin B12 levels and age; nevertheless, it is important to note that cross-sectional studies inherently lack the ability to demonstrate causality. Furthermore, the assessment of vitamin B12 status solely relied on the examination of serum vitamin B12 levels, with no consideration given to the evaluation of homocysteine levels.

## Conclusions

In summary, the findings of the present investigation indicate that there is no significant correlation between vitamin B12 levels and the presence of obesity and diabetes mellitus. Our research offers significant contributions to understanding the interplay among obesity, diabetes, and B12 levels. The relationship between vitamin B12 and obesity is a versatile and developing field of research. However, it is evident that these associations are intricate and subject to various external influences, such as dietary patterns, supplement usage, and underlying metabolic determinants. Subsequent investigations of broader magnitude and enhanced dietary regulations have the potential to provide additional insights into these associations.

## References

[1] Huđek Turković A, Matovinović M, Žuna K, Škara L, Kazazić S, Bačun-Družina V, Durgo K (2022). Association of vitamins D, B(9) and B(12) with obesity-related diseases and oral microbiota composition in obese women in Croatia. Food Technol Biotechnol.

[2] Klisić A, Kavarić N, Spasojević-Kalimanovska V, Kotur-Stevuljević J, Ninić A (2021). Serum endocan levels in relation to traditional and non-traditional anthropometric indices in adult population. J Med Biochem.

[3] Boachie J, Adaikalakoteswari A, Samavat J, Saravanan P (2020). Low vitamin B12 and lipid metabolism: evidence from pre-clinical and clinical studies. Nutrients.

[4] Thomas-Valdés S, Tostes MD, Anunciação PC, da Silva BP, Sant'Ana HM (2017). Association between vitamin deficiency and metabolic disorders related to obesity. Crit Rev Food Sci Nutr.

[5] Geiker NR, Veller M, Kjoelbaek L (2018). Effect of low energy diet for eight weeks to adults with overweight or obesity on folate, retinol, vitamin B(12), D and E status and the degree of inflammation: a post hoc analysis of a randomized intervention trial. Nutr Metab (Lond).

[6] Adaikalakoteswari A, Wood C, Mina TH (2020). Vitamin B12 deficiency and altered one-carbon metabolites in early pregnancy is associated with maternal obesity and dyslipidaemia. Sci Rep.

[7] Hasbaoui BE, Mebrouk N, Saghir S, Yajouri AE, Abilkassem R, Agadr A (2021). Vitamin B12 deficiency: case report and review of literature. Pan Afr Med J.

[8] Al-Maskari MY, Waly MI, Ali A, Al-Shuaibi YS, Ouhtit A (2012). Folate and vitamin B12 deficiency and hyperhomocysteinemia promote oxidative stress in adult type 2 diabetes. Nutrition.

[9] Kaya C, Cengiz SD, Satiroğlu H (2009). Obesity and insulin resistance associated with lower plasma vitamin B12 in PCOS. Reprod Biomed Online.

[10] Kibirige D, Mwebaze R (2013). Vitamin B12 deficiency among patients with diabetes mellitus: is routine screening and supplementation justified?. J Diabetes Metab Disord.

[11] Tavares Bello C, Capitão RM, Sequeira Duarte J, Azinheira J, Vasconcelos C (2017). Vitamin B12 deficiency in type 2 diabetes mellitus. Acta Med Port.

[12] Li Z, Gueant-Rodriguez RM, Quilliot D, Sirveaux MA, Meyre D, Gueant JL, Brunaud L (2018). Folate and vitamin B12 status is associated with insulin resistance and metabolic syndrome in morbid obesity. Clin Nutr.

[13] Reinstatler L, Qi YP, Williamson RS, Garn JV, Oakley GP Jr (2012). Association of biochemical B₁₂ deficiency with metformin therapy and vitamin B₁₂ supplements: the National Health and Nutrition Examination Survey, 1999-2006. Diabetes Care.

[14] Monasso GS, Santos S, Geurtsen ML, Heil SG, Felix JF, Jaddoe VW (2021). Associations of early pregnancy and neonatal circulating folate, vitamin B-12, and homocysteine concentrations with cardiometabolic risk factors in children at 10 y of Age. J Nutr.

[15] O'Malley EG, Reynolds CM, Cawley S, Woodside JV, Molloy AM, Turner MJ (2018). Folate and vitamin B12 levels in early pregnancy and maternal obesity. Eur J Obstet Gynecol Reprod Biol.

[16] Moen GH, Qvigstad E, Birkeland KI, Evans DM, Sommer C (2018). Are serum concentrations of vitamin B-12 causally related to cardiometabolic risk factors and disease? A Mendelian randomization study. Am J Clin Nutr.

[17] van Weelden W, Seed PT, Antoun E (2022). Folate and vitamin B12 status: associations with maternal glucose and neonatal DNA methylation sites related to dysglycaemia, in pregnant women with obesity. J Dev Orig Health Dis.

[18] Gunanti IR, Marks GC, Al-Mamun A, Long KZ (2014). Low serum vitamin B-12 and folate concentrations and low thiamin and riboflavin intakes are inversely associated with greater adiposity in Mexican American children. J Nutr.

[19] Sukumar N, Venkataraman H, Wilson S, Goljan I, Selvamoni S, Patel V, Saravanan P (2016). Vitamin B12 status among pregnant women in the UK and its association with obesity and gestational diabetes. Nutrients.

[20] Aroda VR, Edelstein SL, Goldberg RB (2016). Long-term metformin use and vitamin B12 deficiency in the diabetes prevention program outcomes study. J Clin Endocrinol Metab.

[21] Ray JG, Wyatt PR, Thompson MD (2007). Vitamin B12 and the risk of neural tube defects in a folic-acid-fortified population. Epidemiology.

[22] Chakraborty S, Chopra M, Mani K (2018). Prevalence of vitamin B(12) deficiency in healthy Indian school-going adolescents from rural and urban localities and its relationship with various anthropometric indices: a cross-sectional study. J Hum Nutr Diet.

[23] Ciftel S, Bilen A, Yanıkoglu ND (2022). Vitamin B12, folic acid, vitamin D, iron, ferritin, magnesium, and HbA1c levels in patients with diabetes mellitus and dental prosthesis. Eur Rev Med Pharmacol Sci.

[24] Stabler SP (2013). Clinical practice. Vitamin B12 deficiency. N Engl J Med.

[25] Jayashri R, Venkatesan U, Rohan M (2018). Prevalence of vitamin B(12) deficiency in South Indians with different grades of glucose tolerance. Acta Diabetol.

[26] Owen MD, Baker BC, Scott EM, Forbes K (2021). Interaction between metformin, folate and vitamin b12 and the potential impact on fetal growth and long-term metabolic health in diabetic pregnancies. Int J Mol Sci.

[27] de Jager J, Kooy A, Lehert P (2010). Long term treatment with metformin in patients with type 2 diabetes and risk of vitamin B-12 deficiency: randomised placebo controlled trial. BMJ.

[28] Green R, Allen LH, Bjørke-Monsen AL (2017). Correction: vitamin B(12) deficiency. Nat Rev Dis Primers.

[29] Johnson MA (2007). If high folic acid aggravates vitamin B12 deficiency what should be done about it?. Nutr Rev.

